# Salivary caffeine in Parkinson’s disease

**DOI:** 10.1038/s41598-021-89168-6

**Published:** 2021-05-10

**Authors:** Giorgio Leodori, Maria Ilenia De Bartolo, Daniele Belvisi, Alessia Ciogli, Andrea Fabbrini, Matteo Costanzo, Simone Manetto, Antonella Conte, Claudio Villani, Giovanni Fabbrini, Alfredo Berardelli

**Affiliations:** 1 IRCCS NEUROMED, Via Atinense, 18, 86077 Pozzilli, Italy; 2 Department of Chemistry and Drug Technologies, Sapienza University of Rome, Piazzale Aldo Moro 5, 00185 Rome, Italy; 3 Department of Human Neurosciences, Sapienza University of Rome, Viale dell’Università 30, 00185 Rome, Italy

**Keywords:** Neuroscience, Diseases of the nervous system, Neurology, Neurological disorders

## Abstract

We aimed to investigate salivary caffeine content, caffeine absorption and metabolism in Parkinson’s disease (PD) and verify whether salivary caffeine can be used as a biomarker of PD. We enrolled 98 PD patients and 92 healthy subjects. Caffeine and its major metabolite, paraxanthine, were measured in saliva samples collected before and 4 h after the oral intake of caffeine (100 mg). We measured caffeine absorption as the normalized increase in caffeine levels, and caffeine metabolism as the paraxanthine/caffeine ratio. The Movement Disorder Society Unified Parkinson's Disease Rating Scale part III, the Hoehn & Yahr, the presence of motor complications, and levodopa equivalent dose (LED) were assessed and correlated with caffeine levels, absorption, and metabolism. The effects of demographic and environmental features possibly influencing caffeine levels were also investigated. Caffeine levels were decreased in patients with moderate/advanced PD, while caffeine levels were normal in patients with early and de-novo PD, unrelated to caffeine intake. Caffeine absorption and metabolism were normal in PD. Decreased salivary caffeine levels in PD were associated with higher disease severity, longer duration, and the presence of motor complications, no significant association was found with LED. Salivary caffeine decrease correlates with PD progression.

## Introduction

Previous studies have demonstrated that caffeine may be involved in the pathophysiology of Parkinson’s disease (PD) ^1–4^. One case–control study in a Japanese cohort found decreased serum caffeine in a population of moderate-advanced PD patients as compared to healthy subjects (HS), with no differences in CYP1A2 genotype, which is the enzyme responsible for 95% of caffeine metabolism to paraxanthine, or caffeine intake ^5^. However, it is still unknown whether caffeine is reduced even in early patients not on antiparkinsonian medications (de novo PD patients), and whether PD patients consume or metabolize caffeine differently from HS.

Caffeine levels and metabolism can be investigated through blood as well as saliva samples ^6–11^. One study using saliva samples found that PD patients had decreased caffeine levels but normal caffeine metabolism ^12^. However, this study did not consider several important environmental and demographic confounders, such as caffeine intake, and did not investigate relationships between caffeine levels and PD clinical features.

Since saliva testing provides an easy and noninvasive biospecimen, it would first be important to see whether caffeine is reduced in the saliva of PD patients, and then investigate caffeine metabolism after having controlled for relevant environmental and demographic factors. Finally, it is unknown whether caffeine levels correlate with clinical features in PD patients.

Therefore, we investigated salivary caffeine levels in PD patients and compared the results with those of age-matched HS in a Caucasian population. We also studied caffeine absorption and metabolism after the oral intake of caffeine. Furthermore, we investigated whether caffeine levels, absorption, and metabolism correlate with clinical features of PD, and the possible influence of demographic and environmental factors.

## Results

A total of 86 PD patients (mean age 65.7 ± 0.94 years, 30 females) and 83 HS (mean age 63.7 ± 1.16 years, 45 females) were included in the analysis. Thirty-nine PD patients were included in the early PD group (including de novo cases) (mean age 61.1 ± 7.33; 24 females) and were compared to 39 age-matched HS (mean age 59.6 ± 7.17, 14 females), whereas 47 PD patients were included in the moderate/advanced PD group (mean age 69.71 ± 10.59, 18 females) and were compared to 44 age-matched HS (mean age 67.75 ± 8, 20 females) (Table 1). None of the patients enrolled had underwent surgical interventions for Parkinson’s disease.

**Table 1:** Patient clinical characteristics. Abbreviations: H&Y = Hoehn & Yahr; UPDRS = Unified Parkinson's Disease Rating Scale; LED = levodopa equivalent dose. ^a^Wearing off and/or dyskinesia.

Group	Number	Disease duration (yrs)	H&Y (stage)	UPDRS (part III score)	LED (mg/day)	Motor complications^a^ (yes/no)
Early	39	1.6 ± 0.9	1.4 ± 0.4	14.6 ± 5.8	150.1 ± 151.3	0/39
Moderate/advanced	47	8.8 ± 6.7	2.5 ± 0.7	27.6 ± 11.6	580.9 ± 214	31/16

All female participants were in menopause and none were assuming estrogen-progestin therapies. In PD patients and HS, daily caffeine intake significantly predicted basal caffeine concentration (F_(1,165)_ = 7.633, p < 0.01, adj. R2 = 3.8%; B = 0.82, SEb = 0.29; caffeine intake: B = 0.003, SEb = 0.001, β = 0.21). In all participants heavy coffee consumption was associated with higher CYP1A2 activity (0.30 ± 0.19 vs. 0.26 ± 0.34, U = 4344, p < 0.01). There was a significant increase in salivary caffeine concentration at T1 (2.13 ± 1.75 μg/ml) compared to T0 (1.54 ± 1.74) (z = 5.73, p < 0.01). Finally, we found no significant correlation between age and basal caffeine concentration (τb = -0.023, p = 0.668), caffeine absorption (τb = -0.036, p = 0.502) and CYP1A2 activity (τb = 0.069, p = 0.197).

### Salivary caffeine in PD patients and HS

When comparing the PD and HS groups as a whole, there were no significant differences in basal caffeine levels (1.43 ± 1.65 vs. 1.66 ± 1.80 μg/ml; U = 837, p = 0.15), caffeine absorption (0.59 ± 1.49 vs. 0.51 ± 1.95 μg/ml; U = 606, p = 0.30), or CYP1A2 activity (0.28 ± 0.26 vs. 0.28 ± 0.29; U = 700, p = 0.98). Similarly, early PD patients and HS showed statistically similar basal caffeine levels (1.81 ± 2.10 vs. 1.11 ± 1.13 μg/ml; U = 837, p = 0.15), caffeine absorption (0.34 ± 1.47 vs. 0.94 ± 1.61 μg/ml; U = 605, p = 0.30), and CYP1A2 activity (0.29 ± 0.28 vs. 0.31 ± 0.36; U = 700, p = 0.98). Conversely, moderate/advanced PD patients compared with HS showed significantly lower levels of basal caffeine (1.13 ± 1.11 vs. 2.13 ± 2.14 μg/ml; U = 721, p = 0.009), significantly higher caffeine absorption (0.79 ± 1.48 vs. 0.11 ± 2.14 μg/ml; U = 1348, p = 0.022), and statistically similar CYP1A2 activity (0.26 ± 24 vs. 0.25 ± 0.20; U = 1007, p = 0.83).

### Correlation between salivary caffeine levels and clinical features of PD

**Figure 1:**
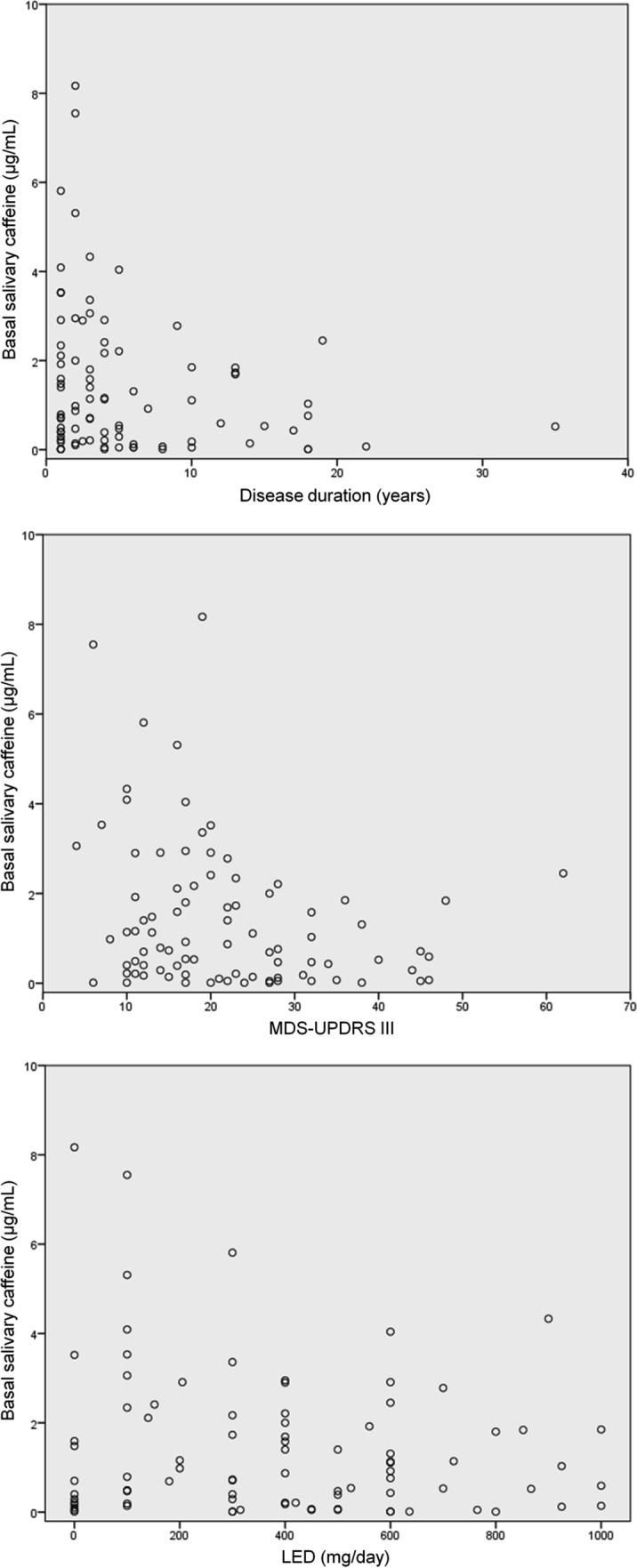
Basal salivary caffeine levels plotted against disease duration (top), Movement Disorder Society-sponsored revision of the Unified Parkinson's Disease Rating Scale (MDS-UPDRS) part III score (medium), and levodopa equivalent dose (LED) (bottom) of all PD patients.

Basal caffeine showed a significant negative association with disease severity (τb = -0.173, p = 0.02) and disease duration (τb = -0.154, p = 0.04), and a non-significant negative correlation with LED (τb = -0.066, p = 0.39) (Fig. 1).

There was no significant association between caffeine absorption or CYP1A2 activity and disease duration, severity, or LED (all p > 0.05).

In the moderate/advanced PD group, those with motor complications, as compared to those without, had a significantly lower basal salivary caffeine concentration (0.70 ± 0.73 vs. 1.90 ± 1.32 μg/ml, U = 109, p < 0.01), a significantly longer disease duration (11.6 ± 6.8 vs. 3.7 ± 1.6 years, U = 471, p < 0.01), a higher MDS-UPDRS part III score (32.2 ± 11.5 vs. 19.2 ± 5.8, U = 426, p < 0.01), a lower consumption of coffee cups/day (2.1 ± 1.3 vs. 2.7 ± 1.2 cups, U = 156, p = 0.03), and a lower daily caffeine intake (178.06 ± 109.53 mg/day vs. 230.64 ± 102.7, U = 558, p < 0.01), whereas age, BMI, LED, caffeine absorption, and CYP1A2 activity were statistically similar between patients with and without motor complications (all p > 0.05). However, a follow-up ANCOVA showed that even after adjustment for daily caffeine intake, disease duration, and MDS-UPDRS part III score, there was a statistically significant difference in basal salivary caffeine concentration between moderate/advanced PD patients with and without motor complications (F_(1,42)_ = 10.77, p < 0.01).

### Demographic and environmental factors

We found no statistically significant differences in basal caffeine levels, caffeine absorption, or CYP1A2 activity between male and female participants, both when considering the PD and HS groups as a whole and when analyzed in subgroups (Table 2). There were no statistically significant differences between PD patients and HS in terms of age, BMI, coffee cups/day, or caffeine intake, both when considering the groups as a whole and as subgroups (Table 3). Only 15 participants reported tobacco smoking (6 HS, 4 early, and 5 moderate/advanced PD patients), and only 9 PD patients were taking ropinirole (4 early and 5 moderate/advanced PD patients). All comparisons between groups yielded similar results when repeated after excluding tobacco smokers and patients taking ropinirole (data available upon request). Twenty-two out of 63 PD patients were taking the CYP1A2-substrate rasagiline, and these patients showed significantly higher basal caffeine levels as compared to those who were not (U = 913, p = 0.03). Rasagiline had no significant effect on caffeine absorption (U = 595, p = 0.33) or CYP1A2 activity (U = 797, p = 0.24). An analysis repeated after excluding the 22 patients taking rasagiline confirmed no statistical difference in basal caffeine levels between PD patients and HS (U = 2195, p = 0.11). We found no significant effect of tobacco smoke, rasagiline, or ropinirole on basal caffeine levels, caffeine absorption, or CYP1A2 activity when early and moderate/advanced PD subgroups were considered separately (all p > 0.05). None of the participants included in the analysis reported a high consumption of foods known to modulate CYP1A2 activity in the 24 h preceding the study. Only one PD patient was taking carbamazepine, which is a known CYP1A2 inducer, and only one HS and one PD patient were using omeprazole, which is reported to be a CYP1A2 inducer too.

**Table 2:** Effect of gender on caffeine level, absorption, and metabolism.

Whole group	Male	Female	p values
Number	94	75	
Basal caffeine level	1.42 ± 1.51	1.68 ± 1.98	0.51
Caffeine absorption	0.46 ± 1.42	0.64 ± 2.04	0.21
CYP1A2 activity	0.27 ± 0.23	0.29 ± 0.31	1.00

Early PD and age-matched HS	Male	Female	p values
Number	40	37	
Basal caffeine level	1.32 ± 1.61	1.61 ± 1.85	0.64
Caffeine absorption	0.41 ± 1.33	0.90 ± 1.79	0.50
CYP1A2 activity	0.25 ± 0.22	0.34 ± 0.40	0.25

Moderate/advanced PD and age-matched HS	Male	Female	p values
Number	54	38	
Basal caffeine level	1.49 ± 1.45	1.77 ± 2.10	0.63
Caffeine absorption	0.50 ± 1.50	0.41 ± 2.28	0.23
CYP1A2 activity	0.28 ± 0.25	0.22 ± 0.17	0.26

**Table 3:** Participant demographic and environmental characteristics.

	PD group as a whole	HS group as a whole	p values
Number	86	83	–
Male/female	56/30	38/45	0.018
Age (yrs, mean ± SD)	65.7 ± 0.94	63.7 ± 1.16	0.10
BMI (mean ± SD)	25.32 ± 4.17	25.04 ± 3.34	0.99
Coffee cups/day (mean ± SD)	2.53 ± 0.15	2.66 ± 0.15	0.53
Caffeine intake, mg/day (mean ± SD)	211.69 ± 107.58	217.81 ± 110.18	0.77
Tobacco smoking (n)	9	6	0.34

	Early PD	Age-matched HS	p values
Number	39	39	–
Male/female	15:24	25:14	< 0.01
Age (yrs, mean ± SD)	61.1 ± 7.33	59.6 ± 7.17	0.16
BMI (mean ± SD)	24.33 ± 4.06	24.45 ± 3.20;	0.51
Coffee cups/day (mean ± SD)	2.79 ± 1.34	2.64 ± 1.44	0.72
Caffeine intake, mg/day (mean ± SD)	234.10 ± 104.60	211.89 ± 113.08	0.46
Tobacco smoking	4	3	0.89

	Moderate/advanced PD	Age-matched HS	p values
Number	47	44	–
Male/female	29:18	24:20	0.66
Age (yrs, mean ± SD)	69.71 ± 10.59	67.75 ± 8.00	0.13
BMI (mean ± SD)	26.31 ± 4.11	25.50 ± 3.44	0.47
Coffee cups/day (mean ± SD)	2.32 ± 1.33	2.69 ± 1.29	0.23
Caffeine intake, mg/day (mean ± SD)	193.09 ± 107.55	223.84 ± 108.72	0.25
Tobacco smoking	5	3	0.46

## Discussion

In the present study, we investigated salivary caffeine levels, caffeine absorption, and caffeine metabolism in PD patients with different disease severity and demographics as compared to HS. The first finding was that moderate/advanced PD patients had decreased salivary caffeine levels in comparison to HS, whereas early PD patients, including de novo PD patients, did not. We also found no evidence of reduced caffeine absorption or metabolism in PD patients. There was a negative association between salivary caffeine levels and disease duration and severity, whereas no significant association was found with antiparkinsonian therapy. PD patients with motor complications had significantly lower basal salivary caffeine levels as compared to patients without motor complications, independent of daily caffeine intake, disease duration, and severity. Finally, results were controlled for age, gender, BMI, daily caffeine intake, tobacco smoking, and other foods and medications known to modulate caffeine metabolism.

Our findings that daily caffeine intake significantly determined caffeine levels, that subjects with higher coffee consumption had increased caffeine metabolism, and that caffeine levels were significantly higher after the administration of caffeine all confirmed the accuracy of the methods we used. Since we also studied de novo patients, we were able to verify whether caffeine was already reduced in the early stages of the disease before the introduction of any antiparkinsonian medication.

Caffeine levels were controlled for most of the main demographic and environmental factors known to affect caffeine levels and metabolism since PD patients and HS did not differ in terms of age, BMI, coffee consumption, or total daily caffeine intake. Gender had no significant effect on salivary caffeine levels, caffeine absorption, or caffeine metabolism. Furthermore, our results were not significantly affected by CYP1A2 modulators, including tobacco smoking, rasagiline, and ropinirole, and the number of participants taking other CYP1A2-modulating medications and food was negligible. Since we found that LED was not associated with caffeine levels, metabolism, and absorption, we exclude that antiparkinsonian therapy significantly affected our results.

The cause of decreased salivary caffeine concentrations in PD patients is unclear. Fujimaki et al. ^5^ suggested that the intestinal absorption of caffeine may be impaired in PD. However, we found that PD patients and HS had similar caffeine absorption and metabolism. Another study investigated CYP1A2 in saliva samples in PD patients and found decreased baseline caffeine levels but normal caffeine metabolism ^12^. However, they did not investigate several important environmental and demographic confounders, such as daily caffeine intake, and possible relationships between caffeine levels and PD clinical features. The binding of caffeine to plasmatic albumin is around 27–30% ^13^. A previous study found that decreased serum albumin is an independent risk factor for PD and that PD progression is associated with a progressive reduction in albumin levels ^14^. The reduction in albumin levels as PD progresses may lead to a higher proportion of free caffeine subject to faster metabolism, thus resulting in lower caffeine concentrations ^15^.

The observation that caffeine levels were lower only in moderate/advanced PD is partially in contrast with that of Fujimaki et al. ^5^ who found decreased caffeine even in early stages of PD. Since the pharmacokinetics of salivary caffeine accurately and linearly reflects that in the blood ^9–11^, differences in the biospecimen used between the two studies cannot explain the different results. Differences in the patient populations studied might explain the differences between the present study (which also included de novo patients) and the study of Fujimaki et al. ^5^ who did not include de novo patients. Other explanations include the known differences in CYP1A2 activity between subjects of South Asian and European ancestry ^16^, and the lower mean caffeine intake reported by Fujimaki et al. ^5^ in both patients and controls compared to that found in our study population and in a larger Japanese cohort ^17^.

The result that salivary caffeine was only reduced in moderate/advanced PD patients, and that disease duration and severity correlated with decreased caffeine levels strongly support the hypothesis that caffeine levels in PD are a marker of disease progression^18,19^. This hypothesis is also supported by our finding that the presence of motor complications was associated with lower basal caffeine levels, even after adjusting for caffeine intake, disease duration, and severity. The hypothesis that caffeine decreases as PD progresses needs to be confirmed by longitudinal studies. Also, in a progressive rat model of PD, chronic caffeine consumption has shown to be able to prevent dopaminergic degeneration in the substantia nigra even when caffeine was introduced after the onset of the neurodegenerative process ^20^. In the light of the above observation, longitudinal studies may also verify whether salivary caffeine is a predictor of disease progression ^21^. Furthermore, it would be important to correlate salivary caffeine with other salivary markers linked to Parkinson's disease pathology ^22–24^.

We acknowledge that our study has some limitations. Disease severity was assessed with the MDS-UPDRS score and while the patients were on medications. The assessment of patients under medication might had reduced the between-patients variability of disease severity producing in the correlation analysis a possible bias leading to false negative results (type II error). The correlation analysis performed on the entire PD population showed that the decrease in salivary caffeine was significantly associated with disease severity, and also with disease duration and the presence of motor complications. Therefore, we excluded that a type II error significantly affected the correlation analysis, and our results therefore provide evidence for an association between decreased caffeine levels and PD progression. We cannot exclude that a larger group of early PD patients might have unveiled a reduced level of caffeine in this population. Slowed gastric emptying may have delayed the caffeine concentration peak in PD patients thus biasing our results of caffeine metabolism and absorption. However, previous studies using gold-standard techniques found no evidence of slowed gastric emptying in PD ^25^. Although the lack of a significant number of smokers in our study population excluded possible confounding due to CYP1A2 induction ^13^, it also prevented us from drawing conclusions regarding the effect of tobacco smoke on caffeine levels in PD. We investigated caffeine absorption only by means of an indirect method, and therefore further studies with direct quantification are needed to rule out abnormal caffeine absorption in PD. A final comment concerns our finding that coffee consume was similar between PD patients and HS is in contrast with previous observations that PD patients consume less coffee than HS. Larger cohorts are probably needed to highlight this epidemiological difference ^3^.

In conclusion, our study demonstrates that salivary caffeine levels are lower in moderate/advanced PD patients than in HS and reduced caffeine levels are not due to abnormal caffeine absorption and metabolism. The correlation we found between salivary caffeine levels and PD severity and duration suggests that salivary caffeine may be a biomarker of disease progression that provides new insight into PD pathogenesis.

## Methods

### Participants

We consecutively enrolled 98 PD patients (mean age 66 ± 4; 36 females) from the movement disorders outpatient unit at the Department of Human Neuroscience, Sapienza University of Rome. Data obtained in patients were compared with a group of 92 HS (mean age 64 ± 11; 48 females) frequency-matched for age with PD patients. All enrolled subjects agreed to participate in the study. This study was conducted between October 2018 and December 2019. Inclusion criteria were age > 18 years and a diagnosis of PD based on international criteria ^26^. Exclusion criteria were: pregnancy and breast-feeding; caffeine intolerance; the diagnosis of other neurological or psychiatric conditions; serious concomitant medical conditions (e.g. congestive heart failure/NYHA > 2, arrhythmias, severe chronic obstructive pulmonary disease, autoimmune diseases, neoplasms, chemotherapy); the use of illicit drugs in the past 6 months; or the consumption of more than 7 alcoholic drinks/week for females and more than 14 for males. All enrolled participants were informed about the study objectives. Coffee consumption habits did not influence any participant’s decision on whether or not to participate in the study.

The Movement Disorder Society-sponsored revision of the Unified Parkinson's Disease Rating Scale (MDS-UPDRS) part III was used to assess motor symptom severity and was performed with patients on pharmacological treatment, about one hour after each patient’s regular dose of antiparkinsonian medications taken in the morning ^27^. The stage of the disease was assessed by means of the Hoehn and Yahr scale (H&Y) ^28^. Disease duration was calculated from symptom onset. The presence of motor complications (wearing off and/or dyskinesia) was also assessed with the MDS-UPDRS part IV. The levodopa equivalent dose (LED) was calculated according to Tomlinson et al. ^29^. Patients were also divided into two groups: the early PD group included patients with a disease duration ≤ 3 years and an H&Y score ≤ 2, and also included 12 patients who were de novo (i.e. patients with a disease duration shorter than 2 years and drug naïve for antiparkinsonian medications), whereas the moderate/advanced PD group included patients with a disease duration > 3 years and/or an H&Y score ≥ 2. Each PD group was compared with an age-matched control group. HS were chosen from non-consanguineous relatives of PD patients. We collected information regarding possible CYP1A2 modulators, including tobacco smoke, medications, estrogen-progestin therapies, the presence or absence of menopause in enrolled females, food consumed in the past 24 h, and ethnic origin [16,30,]. As possible confounding factors known to modulate caffeine metabolism, we calculated body mass index (BMI) and daily caffeine intake during the last year (cups/day and mg/day) by considering the following values: coffee (espresso)/80 mg, cup of tea (220 ml)/50 mg, can of cola (355 ml)/40 mg, chocolate bar (50 g)/25 mg (http://www.efsa.europa.eu/en/microstrategy/food-composition-data). The study protocol was approved by the institutional review board of Sapienza University of Rome (study 503/18 approved 25 June 2018) and conducted in accordance with the latest revision of the Declaration of Helsinki. All participants gave their written informed consent prior to participating in the study.

### Sample collection

Participants were instructed not to consume caffeine or caffeinated products for at least 12 h prior to the test. Patients were also instructed to assume their usual medications. All participants were tested at approximately the same time of day after fasting overnight (the first saliva sample was collected between 8:00 and 11:00 AM).

Saliva samples (2-3 ml) were collected in plain 5-ml tubes a few minutes before (T0) and 4 h after (T1) the assumption of a capsule containing 100 mg of caffeine (hospital pharmacy). Participants were asked not to drink in the hour before samples collection, and to fast during the 4 h between T0 and T1. Saliva samples were stored at -20 degrees until the time of analysis.

### Sample analysis

Acetonitrile (HPLC gradient grade), water (HPLC gradient grade), formic acid (reagent grade), sodium phosphate monobasic (ACS reagent ≥ 99%), 2-propanol (HPLC gradient grade), caffeine (anhydrous 99%), benzotriazole (ReagentPlus 99%), paraxanthine (98%) were purchased from Sigma Aldrich (St. Louis, MO). The SPE was performed by using Oasis HLB 96-well plate (30 mg/well) and a 96-well extraction vacuum manifold (Waters Corporation, Milford, MA, USA). Standard stock solutions of caffeine, paraxanthine and benzotriazole were prepared in water (HPLC-gradient grade).

Experiments were carried out on an Accela UHPLC System Thermo Fisher Scientific (San Jose, CA) which consisted of an Accela 1250 Pump, an Accela autosampler and an Accela PDA photodiode array detector, operated at 270 nm. Chromatographic data were collected and processed using the Thermo Xcalibur Chromatography Manager software, version 1.0. A guard cartridge system (SecurityGuard Ultra UHPLC) has been connected to an analytical column Kinetex 2.6 µm EVO C18 100 Å 100 × 3.0 mm (L. x I.D.), both from Phenomenex, Torrance, CA, USA. All analyses were performed at 25 °C, the mobile phase was a mixture of water and acetonitrile (95:5% *v/v*) with 0.1% formic acid, and it was filtered through 0.2 µm Omnipore filters (Merck Millipore, Darmstadt, Germany) before use. Injected volumes were 10 µL. The mobile phase was delivered at a total flow rate of 0.5 mL/min. The total run time was 10.0 min, and under these conditions, paraxanthine, caffeine and benzotriazole had a retention times of 1.62, 2.72 and 3.65 min respectively.

Concisely, 500 µL of aqueous sodium phosphate monobasic solution (50 mM) and 100 µL of aqueous benzotriazole solution (80 µg/mL) were added to 500 µL of saliva.

Afterwards, the sample was loaded into the SPE cartridge, followed by a washing step with 1 mL HPLC grade water. The adsorbed caffeine, paraxanthine and benzotriazole were eluted with 1 mL of an acetonitrile / IPA solution 90:10% *v/v*. The SPE was performed by using Oasis HLB 96-well plate (30 mg/well) and a 96-well extraction vacuum manifold (Waters Corporation, Milford, MA, USA). The eluate was dried under nitrogen flow at a temperature of 35 °C, taken up with 1 mL of HPLC water and transferred to HPLC vials.

Quantitative analysis was performed using internal standard calibration method. Standard stock solutions of caffeine, paraxanthine and benzotriazole of 1 mg/mL were prepared in water (HPLC-gradient grade) stored in glass bottles with screw caps at 4 °C and were prepared freshly with every batch of analyses. Method validation was achieved evaluating linearity, limit of detection (LOD), limit of quantification (LOQ), specificity, selectivity*,* precision and accuracy.

Calibration curves in blank matrix (saliva sample was obtained from healthy volunteer following abstinence from all caffeine-containing products) and in aqueous solution were prepared in triplicate. Caffeine and paraxanthine were added in order to obtain concentrations of 0.075, 0.25, 0.5, 1, 2.5, 5, 10, 12.5, 20, 25 µg/mL, in addition benzotriazole (8 µg/mL) was used as internal standard. Calibration curves were obtained by using least-squares linear regression model: the ratio of analyte /internal standard peak-area was plotted as a function of analyte concentration. For caffeine and paraxanthine a correlation coefficient (R^2^) of 0.998 was determined for both analytes in saliva. Moreover, the slope of the calibration curves showed no influence by the matrix. The limit of detection (LOD) and the limit of quantification (LOQ) were determined by measuring a mixture of standard solutions until a signal-to-noise ratio of 3 (S/N = 3) for LOD, and a signal-to-noise ratio of 10 (S/N = 10) for LOQ was reached. For both analytes the value of LOD was 0.075 µg/mL, whereas the value of the LOQ was 0.25 µg/mL.

The coelution with endogenous compounds was excluded by the analysis of several blank matrices.

The intra-day and inter-day precision and accuracy (percentage of coefficient of variation %CV) for caffeine and paraxanthine in saliva was considered at three concentrations, which covered the range of measurement. The precision and accuracy for both caffeine and paraxanthine in saliva were within the acceptable range (< 15%).

The recovery was reproducible, for both caffeine (96.0 ± 3.0%) and paraxanthine (94.3 ± 3.8%). The recovery for the internal standard (benzotriazole) was 91.1 ± 1.8%.

Samples from 12 out of the 98 PD patients and 11 out of the 92 HS could not be analyzed for technical reasons independent of caffeine levels or PD characteristics. These subjects were excluded from the analysis.

### Statistical analyses

Statistical analyses were performed using SPSS Statistics (25.0.0; IBM). Caffeine absorption was defined as the normalized difference in caffeine concentrations between pre (T0) and post (T1) caffeine intake [(T1—T0)/(T0)], and CYP1A2 activity/ “phenotype” was computed as the ratio between paraxanthine and caffeine concentrations at T1 ^31,32^. We first evaluated by using linear regression the effect of daily caffeine intake on caffeine levels and metabolism. We then used Wilcoxon signed-rank tests to assess whether heavy coffee consumption, defined as ≥ 3 cups/day affected CYP1A2 activity, and whether caffeine concentration increased after oral intake. Separate Mann–Whitney U tests were run to determine if there were differences in basal caffeine levels, absorption, or metabolism between HS and PD patients as a whole, or between early and moderate/advanced PD patients vs. the corresponding HS subgroups. We then assessed whether the outcome measures differed between patients with and without motor complications in the moderate/advanced PD group. Mann–Whitney U tests were also performed to investigate differences in age, BMI score, and daily caffeine intake between the groups as a whole and subgroups, and to investigate the effects of gender, tobacco smoke, and the CYP1A2-substrates rasagiline and ropinirole. We investigated differences in male/female and smokers/non-smokers proportions with the chi-square test of homogeneity. Follow-up covariate-controlled analyses were run when required. Kendall's tau-b correlation coefficient was used to determine the relationship between each of the three dependent variables, i.e. baseline caffeine levels, caffeine absorption, and metabolism, and disease severity (MDS-UPDRS part III), disease duration (years), and LED. Also, in order to test whether age may affect differences between groups we investigated possible correlations with the dependent variables. Since correlations were limited to three clinical variables defined prior to the study, we did not correct for multiple comparisons ^33^. All caffeine concentrations are expressed as μg/mL. All values are expressed as mean ± standard deviation. Outliers were defined as data points exceeding 2.2 times the interquartile values ^34^. There were three outliers for basal caffeine, seven outliers for caffeine levels at T1, and six outliers for CYP1A2 activity. All outliers were kept in the analyses since the 5% trimmed means were similar to the distribution means and all comparisons repeated without the outliers yielded similar results.

### Power computation

Caffeine concentration mean and standard deviation in PD patients and matched HS is reported to be respectively 23.53 ± 22.4 pmol/10 μl and 79.10 ± 91.5 pmol/10 μl (effect size of 0.83) ^5^. Based on this, it was estimated that 26 subjects in each group would be required to detect a significant difference in baseline caffeine levels between two unpaired samples (one tail) with a power of 90% at a significance level of α = 0.05. The interindividual variability of caffeine elimination is reported to 79% ^35^. Based on this, it was estimated that 83 subjects in each group would be required to detect a 40% difference in caffeine metabolism between two unpaired samples with a power of 90% at a significance level of α = 0.05.

## Data Availability

The datasets generated during and/or analysed during the current study are available from the corresponding author on reasonable request.
